# Gait characteristics and their discriminative power in geriatric patients with and without cognitive impairment

**DOI:** 10.1186/s12984-017-0297-z

**Published:** 2017-08-15

**Authors:** Lisette H. J. C. Kikkert, Nicolas Vuillerme, Jos P. van Campen, Bregje A. Appels, Tibor Hortobágyi, Claudine J. Lamoth

**Affiliations:** 1 University of Groningen, University Medical Centre Groningen, Center for Human Movement Sciences, A. Deusinglaan 1, 9700 AD Groningen, The Netherlands; 2 Université Grenoble Alpes, EA AGEIS, Grenoble, France; 3 Institut Universitaire de France, Paris, France; 4 Department of Geriatric Medicine, MC Slotervaart Hospital, Amsterdam, The Netherlands; 5 Department of Medical Psychology and Hospital Psychiatry, MC Slotervaart Hospital, Amsterdam, The Netherlands

**Keywords:** Gait dynamics, Cognitive impairment, Multivariate analysis, IPod touch, Trunk accelerations, Discriminant analysis, Geriatric patients

## Abstract

**Background:**

A detailed gait analysis (e.g., measures related to speed, self-affinity, stability, and variability) can help to unravel the underlying causes of gait dysfunction, and identify cognitive impairment. However, because geriatric patients present with multiple conditions that also affect gait, results from healthy old adults cannot easily be extrapolated to geriatric patients. Hence, we (1) quantified gait outcomes based on dynamical systems theory, and (2) determined their discriminative power in three groups: healthy old adults, geriatric patients with- and geriatric patients without cognitive impairment.

**Methods:**

For the present cross-sectional study, 25 healthy old adults recruited from community (65 ± 5.5 years), and 70 geriatric patients with (*n* = 39) and without (*n* = 31) cognitive impairment from the geriatric dayclinic of the MC Slotervaart hospital in Amsterdam (80 ± 6.6 years) were included. Participants walked for 3 min during single- and dual-tasking at self-selected speed while 3D trunk accelerations were registered with an IPod touch G4. We quantified 23 gait outcomes that reflect multiple gait aspects. A multivariate model was built using Partial Least Square- Discriminant Analysis (PLS-DA) that best modelled participant group from gait outcomes.

**Results:**

For single-task walking, the PLS-DA model consisted of 4 Latent Variables that explained 63 and 41% of the variance in gait outcomes and group, respectively. Outcomes related to speed, regularity, predictability, and stability of trunk accelerations revealed with the highest discriminative power (VIP > 1). A high proportion of healthy old adults (96 and 93% for single- and dual-task, respectively) was correctly classified based on the gait outcomes. The discrimination of geriatric patients with and without cognitive impairment was poor, with 57% (single-task) and 64% (dual-task) of the patients misclassified.

**Conclusions:**

While geriatric patients vs. healthy old adults walked slower, and less regular, predictable, and stable, we found no differences in gait between geriatric patients with and without cognitive impairment. The effects of multiple comorbidities on geriatric patients’ gait possibly causes a ‘floor-effect’, with no room for further deterioration when patients develop cognitive impairment. An accurate identification of cognitive status thus necessitates a multifactorial approach.

## Background

Even healthy aging modifies gait. Declines in muscle mass and quality, decreased sensory functions, reductions in muscle activation, and a reorganization of the cortical and spinal circuits controlling posture and gait underlie the age-related evolution of gait slowing and abnormalities [1, 2]. Indeed, habitual gait speed decreases as much as 16% per decade, starting at age 60 [3]. Because a slow gait speed predicts numerous clinical conditions later in life [4], gait speed is perhaps the most studied feature of physical performance affected by age.

Notwithstanding the attractiveness of gait speed as a simple summary index of mobility, numerous other characteristics of gait have been established to quantify and diagnose age- and pathology-related gait abnormalities. For example, stride-to-stride variability quantified by the Coefficient of Variation (CoV) is 5.3% in elderly fallers compared to 1.1% in age-matched non-fallers [5]. Measures derived from trunk acceleration signals can be used to characterize postural control during walking and standing by means of quantifying the frequency content, amplitude, self-affinity, variability, synchronization, regularity, and local stability of the lower trunk [6–8]. In the present study, we use the term ‘gait dynamics’, and refer to outcomes that are indicative of the dynamic nature of the gait pattern and reflect overall gait coordination, adaptability, and the ability to accurately respond to perturbations. Gait dynamics can discriminate young and old adults [9], individuals with and without a clinical condition [10], fallers and non-fallers [8, 11–14], and older adults with and without cognitive disorders [15, 16].

However, the results of the latter studies cannot easily be extrapolated to geriatric patients who typically suffer from many clinical conditions that also interact with gait performance. Among many, sarcopenia [17], thoracic kyphosis [10], polypharmacy [18], and cognitive impairment [19] can individually and in combination negatively affect gait. We therefore suspect that different combinations of gait measures are distinctive for geriatric patients vs. healthy old adults. Considering the abundant evidence supporting the effects of cognitive impairment on gait and its potential validity to serve as an early marker of cognitive impairment [19], we also expect that geriatric patients with additional cognitive impairment present with distinct gait characteristics. However, due to a lack of brain and neurophysiological correlates of specific gait outcomes, it is not yet possible to specify exactly which gait outcome corresponds to a clinical condition. A detailed gait analysis, including dynamic gait measures, can therefore help to unravel the underlying causes of gait dysfunction, and identify and predict clinical conditions. The identification of cognitive impairment could be even more accurate during walking while performing a cognitive dual-task [15, 20]. Because gait and cognitive function partly rely on the same cortical resources [21], performing a cognitive demanding task while walking stresses the system and potentially enlarge the effects of cognitive impairment on gait [15, 20].

Hence, the purpose of the study was to determine gait characteristics in three groups: healthy old adults and geriatric patients with and without cognitive impairment. Our hypothesis is that a detailed gait analysis (e.g., measures related to speed, self-affinity, stability, and variability) will (1) quantify unique gait characteristics of the three groups, and (2) accurately discriminate geriatric patients vs. healthy old adults, and geriatric patients with and without cognitive impairment. We derived gait outcomes from trunk acceleration signals in 3D during single- and dual-task walking. Because certain gait outcomes are inter-related while others are complementary to each other, we performed a Partial Least Square – Discriminant Analysis (PLS-DA). PLS combines principal component and regression analyses and extracts gait features by modelling the covariance structures [22]. By delineating and quantifying the information contained in the dynamics of gait, we can identify gait features that are unique to healthy old adults, and geriatric patients with and without cognitive impairment.

## Methods

### Participants

Seventy patients were recruited from the geriatric outpatient dayclinic of the MC Slotervaart hospital in Amsterdam between January 2015 and July 2016 (mean age 80 ± 6.6; 53% women). Inclusion criteria were: age 65 or older. Exclusion criteria were: (1) inability to walk for at least three minutes without a walking aid, (2) having neurodegenerative disorders other than related to dementia (e.g., Parkinson’s), (3) inability to speak fluently Dutch, and (4) having mobility disability caused by pain or by neurological or orthopaedic conditions, limiting function in one or both legs. The Medical Ethical Committee of the MC Slotervaart Hospital approved the study protocol. Data of an additional group of 25 independently living healthy old adults (mean age 65 ± 5.5), recruited from the community, were also included [9]. The latter group of old adults were carefully questioned about their health, and were excluded if they had a history of orthopaedic, cognitive, or neurological problems, or if they used medication that would affect gait or postural control. Hence, this group can be considered a cognitive and physical healthy control group consisting of relatively young older adults.

Age, height, weight, BMI, The Charlson Comorbidity Index (CCI) [23] and the number of medications used (> 4 denoting polypharmacy), were extracted from medical records. Grip strength of the dominant hand was quantified with a Jamar hand-held dynamometer. Patients were diagnosed for cognitive impairment by a geriatrician and a neuropsychologist based on (1) medical records and (2) cognitive performance on the Mini Mental State Examination (MMSE) (range 0-30) [24] and the 7-min screen (7MS) test. The 7MS assessed memory and executive function using the Benton’s Temporal Orientation (range 0-113), the Enhanced Cued Recall (range 0-16), the animal verbal fluency (range 0-45) and clock-drawing test (range 0-14) [25]. Based on the evaluation of the two clinical experts, patients were categorized as either cognitive impaired or cognitive intact, with the cognitive impaired group including patients with a diagnosis for Mild Cognitive Impairment (MCI) or dementia.

### Procedures and data analysis

Participants walked for three minutes at a self-selected speed on a 10-m long course that was marked with cones, under single- and dual-task conditions. In order to capture long-range patterns in the entire accelerations signals, participants were instructed to keep walking, and make comfortable turns around the cones. When patients did not succeed to walk for 3 min, the longest continuous part of the signal was used for the analysis. A phonetic fluency task was introduced in the dual-task condition, in which participants were asked to name as many words starting with the letters ‘g’, ‘p’, or ‘r’ (one minute per letter) while walking. A cognitive single-task with letters ‘d’, ‘a’, and ‘t’ was used as control condition.

Trunk accelerations were registered with an iPod touch G4 (iOS 6, Apple Inc.; sample frequency ± 100 Hz) that was fixed with a belt near the level of lumbar segment L3. The validity of gait and standing posture parameters from trunk accelerations as indicated by intra-class correlation (ICC) was high (ICC = 0.85–0.99), and test–retest reliability was good (ICC = 0.81–0.97) in old adults, under varying conditions [26]. A custom-made application ‘iMoveDetection’ was used to collect and store acceleration data from the built-in tri-axial accelerometer of the iPod [26]. Anterior-posterior (AP), medio-lateral (ML), and vertical (V) acceleration signals were analysed with custom-made software in MATLAB (version 2014b, The MathWorks Inc.). The signals were detrended, corrected for horizontal tilt, and low-pass filtered (Butterworth filter, 4th order; cut-off frequency 10 Hz).

### Gait outcomes

We computed 23 gait outcomes. Gait speed was calculated by dividing distance walked (m) by time (s). The variability of the amplitude of accelerations was indexed by the Root Mean Square (RMS). The Index of Harmonicity (IH) was calculated as an indicator of smoothness, using the power spectrum of accelerations. The IH was estimated as the cumulative sum of the power spectral density of the fundamental frequency (step frequency), divided by that of the subsequent 9 harmonics. An IH of 1 represents a perfect smooth gait [15].

The Cross-sample Entropy (Cross-SampEn) quantified the degree of synchronization between AP and ML, AP and V, and ML and V accelerations. Cross-SampEn is the negative natural logarithm of the conditional probability that epochs with length *m* that match point-wise in the two related signals, repeat itself for *m + 1* points, within a tolerance of *r* (in the present study *m* = 2 and *r* = 0.2). A Cross-SampEn of 0 reflects perfect synchronization between the signals [27].

Gait regularity and symmetry were calculated for AP and V accelerations using the unbiased autocorrelation function of the acceleration signal. The signal was phase shifted with a window approximating average step and stride time. The first peak in de autocorrelation coefficient function relates to step- and the second to stride regularity. A value of 1 reflects perfect regular steps or strides [28]. The difference between step and stride regularity revealed gait symmetry, with 0 representing a perfect symmetric gait [7].

Multi-scale sample Entropy (Mscale-En) is an indicator of gait predictability. Multi-scale entropy takes the complexity of a system into account by calculating the predictability of a signal over time scales with increasing length. A ‘coarse-graining’ process is applied to the acceleration signals; non-overlapping windows of data points with an increasing length *τ* are constructed, with *τ* representing the time scale with a tolerance of *r* (in the present study *τ* = 7 and *r* = 0.2). A complete predictable signal will adopt a Mscale-En value of 0 [29].

Local stability of trunk acceleration patterns was expressed as the *λ*_max_, i.e., maximal Lyapunov exponent, calculated with the Wolf algorithm as this algorithm is most appropriate to evaluate local dynamic stability from relatively small data sets [30]. For the present study, we used an embedding of *n* = 5 dimensions, with a time delay *τ* of 10 samples (0.1 s). Larger *λ*_max_ indicate greater sensitivity to local perturbations.

Finally, stride frequency variability (FreqVar) was computed from AP accelerations. FreqVar was estimated as the relative fluctuations in phase progression [6].

### Statistical analysis

Differences in participant characteristics were examined with a one-way ANOVA with Bonferroni post-hoc test using SPSS version 24. Significance level was set at *p* < 0.05.

A Partial Least Squares Discriminant Analysis (PLS-DA), using the PLS_toolbox for MatLab (version 3.7.1; Eigenvector Research Inc.) was applied. PLS is a combination of principal component and regression analysis, and can handle data with a large number of highly collinear, inter-related variables (gait outcomes) with relatively few observations (participants) [22]. In contrast to usual regression analysis, PLS allows to study interrelations among multiple, interacting gait outcomes. Such a multivariate analysis thus controls for dependencies among gait outcomes and enables to consider the data in an overarching way. Note that this dealing with multicollinearity is crucial, in particular with respect to gait outcomes (e.g., gait speed and stride time are highly correlated). The PLS-DA model identified the internal covariance structure among gait outcomes (X-matrix) that best modelled group (Y-matrix) by removing common variance and by finding underlying latent variables (LV’s). The optimal number of LV’s was determined with the scree plot [22]. All variables were normalized to unit variance. For a more detailed mathematical explanation we refer to the study of Wold and colleagues [22].

The amount of variance explained of each gait outcome by the LV’s indicated the modelling power of those outcomes in predicting the group. Note that a gait outcome without variation may be completely explained by the model, while this outcome may be unimportant to predict group. The Variable Importance in Projection (VIP) value reflects the importance of each individual gait outcome to the particular group. Gait outcomes with a VIP-score > 1.0 are considered important to the model and have a high discriminative power [22].

Violin plots based on the Kernel density distribution showed the distribution of gait outcomes for the three groups, and revealed peaks, bumps, and valleys in the shape of distributions. The size of the kernels demonstrates the density between individual scores, with a large size reflecting heterogeneity among patients.

## Results

Thirty-nine of 70 geriatric patients were diagnosed with cognitive impairment (56%; 10 patients with dementia and 29 with MCI). Geriatric patients were significantly older (80 ± 6.6 years) than healthy old adults (65 ± 5.5 years). Geriatric patients with and without cognitive impairment presented with 1.8 serious comorbidities on average and met the criterion for polypharmacy (>4). Both geriatric patient groups were comparable for all outcomes (age, BMI, handgrip strength, medication use, number of comorbidities), except for cognitive function. Cognitive impaired geriatric patients performed significantly worse on the MMSE and on all sub-scales of the 7MS (Table 1). In addition, all groups performed significantly different on the cognitive single-task (*p* < 0.00), with a score of 14.2 words/min for healthy old adults, and 10.3 and 7.4 words/min for cognitive intact and cognitive impaired geriatric patients, respectively.

**Table 1:** Characteristics of the 95 participants (mean ± SD)^a^

	Healthy old adults (*n* = 25)	Cognitive intact geriatric patients (*n* = 31)	Cognitive impaired geriatric patients (*n* = 39)
Demographics			
Age (years)	65 ± 5.5	79 ± 5.3	82.0 ± 7.2^b, c^
Height (cm)	168 ± 8.6	167 ± 9.4	166 ± 8.2
Weight (kg)	71.3 ± 12.2	73.3 ± 14.6	68.0 ± 12.5
Body Mass Index	25.0 ± 3.6	26.3 ± 5.3	23.5 ± 6.2
Gait speed single task (m/s)	1.20 ± 0.10	0.88 ± 0.22	0.81 ± 0.22^b, c^
Gait speed dual task (m/s)	1.01 ± 0.12	0.69 ± 0.19	0.68 ± 0.22^b, c^
Cognitive function			
Mini Mental State Examination	N.A.	27.4 ± 2.3	23.9 ± 3.8^d^
Benton’s Temporal Orientation	N.A.	4.2 ± 13.8	17.1 ± 29.9^d^
Enhanced Cued Recall	N.A.	14.9 ± 1.7	10.5 ± 4.4^d^
Clock drawing	N.A.	11.8 ± 1.8	9.8 ± 2.6^d^
Verbal fluency	N.A.	18.2 ± 6.6	12.3 ± 4.1^d^
Geriatric syndromes			
Charlson Comorbidity Index	N.A.	1.9 ± 1.8	1.7 ± 1.3
Handgrip strength (kg)	N.A.	26.3 ± 6.4	26.0 ± 7.2
Medication use (number)	N.A.	6.4 ± 4.1	5.5 ± 3.4

^a^Significance set at 5%. ^b^ significant difference between healthy old adults and geriatric patients; ^c^ significant difference between healthy old adults and cognitive impaired geriatric patients; ^d^ significant difference between geriatric and cognitive impaired geriatric patients. *N.A.* Not applicable

Gait outcomes were computed from 298 ± 50 strides on average (mean walking time 177 s, mean stride frequency 1.76 Hz) for single-task walking. For dual-task walking, outcomes were derived from 297 ± 45 strides on average (mean walking time 185 s, mean stride frequency 1.61 Hz).

### Gait characteristics for the 3 groups

For single-task walking, the PLS-DA model contained 4 LV’s that explained 63 and 41% of the variance in gait outcomes (X) and group (Y), respectively. Gait outcomes are logically grouped and divided over the 4 LV’s, with the first LV explaining most of the variance in X and Y. Similarly, the PLS-DA model for dual-task walking consisted of 4 LV’s, explaining 67 and 38% of the variance in respectively gait outcomes (X) and group (Y). VIP-values per group indicated the importance of the gait outcomes to the particular group. Gait outcomes related to speed, regularity, predictability and stability of trunk accelerations revealed with the highest discriminative power (VIP > 1) for both single- and dual-task walking (Table 2).

**Table 2:** PLS-DA model details during single- and dual-task walking for the three groups^a^

Gait outcome	Variance captured per LV (%)					VIP-values					
						Single-task			Dual-task		
	LV1	LV2	LV3	LV4	Total	HO	CI	CIM	HO	CI	CIM
Gait speed	78	5	1	3	86	**1.4**	0.5	**1.3**	**1.7**	**1.2**	**1.5**
RMS AP	58	4	0	0	62	**1.0**	0.7	0.8	**1.1**	**1.2**	0.9
RMS ML	47	1	12	3	62	**1.3**	0.5	**1.2**	**1.3**	0.9	**1.1**
RMS V	76	0	3	2	81	**2.0**	**1.2**	**1.9**	**2.2**	**1.6**	**1.8**
IH AP	53	1	3	1	58	**1.3**	**0.9**	**1.5**	**1.1**	0.8	0.9
IH ML	0	0	24	8	33	0.2	0.4	0.5	0.1	0.0	0.2
IH V	3	11	11	13	37	0.3	**1.7**	0.2	0.2	0.1	0.1
Cross-SampEn AP-ML	42	19	0	6	68	0.8	**1.2**	0.7	**1.0**	**1.4**	0.8
Cross-SampEn AP-V	34	3	15	20	72	0.8	0.3	0.7	**1.1**	0.7	**1.0**
Cross-SampEn ML-V	30	4	8	19	61	0.7	0.9	0.9	**1.2**	0.8	**1.1**
Step Regularity AP	57	2	8	5	72	**1.0**	0.5	0.9	**1.1**	0.7	0.9
Step Regularity V	77	0	4	0	82	**1.4**	0.3	**1. 3**	**1.3**	**1.0**	**1.0**
Stride Regularity AP	73	0	9	3	85	**1.4**	0.5	**1.3**	**1.3**	0.9	**1.0**
Stride Regularity V	82	0	10	1	93	**1.5**	0.3	**1.3**	**1.5**	**1.1**	**1.1**
Symmetry AP	30	2	11	4	47	0.5	0.8	0.7	0.5	0.3	0.6
Symmetry V	44	3	24	0	71	0.9	0.9	**1.4**	0.9	0.6	0.8
Mscale-En AP	19	1	26	5	51	0.3	0.2	0.7	**1.8**	0.6	**3.6**
Mscale-En ML	0	47	0	5	51	0.7	**3.2**	0.3	0.4	0.6	0.5
Mscale-En V	18	20	16	0	53	**1.2**	**3.5**	**1.0**	**1.0**	**2.4**	**1.4**
max-Lyap AP	23	0	8	14	45	0.7	0.4	0.8	**1.4**	**1.0**	**1.1**
max-Lyap ML	20	20	24	2	66	**1.5**	**2.3**	**1.4**	0.5	0.3	0.6
max-Lyap V	53	4	5	2	64	**1.3**	**1.1**	**1.4**	0.3	**3.5**	0.8
FreqVar AP	35	3	0	3	41	**1.0**	0.7	0.8	0.3	**1.2**	0,4

^a^Explained variance (%) per LV for the single-task model, and VIP-values for healthy old (HO) adults, Cognitive Intact (CI) geriatric and Cognitive Impaired (CIM) geriatric patients during single- and dual task walking. A VIP > 1.0 denotes considerable importance of the gait outcome to the particular group (bold). *LV* latent variable, *VIP* Variable Importance in Projection, *RMS* Root Mean Square, *IH* Index of Harmonicity, *Cross-SampEn* Cross Sample Entropy, *Mscale-En* Multi-scale Entropy, *max-Lyap* maximal Lyapunov Exponent, *FreqVar* Frequency Variability, *AP* Anterior-Posterior, *ML* Medio-Lateral, *V* Vertical

**Fig. 1:**
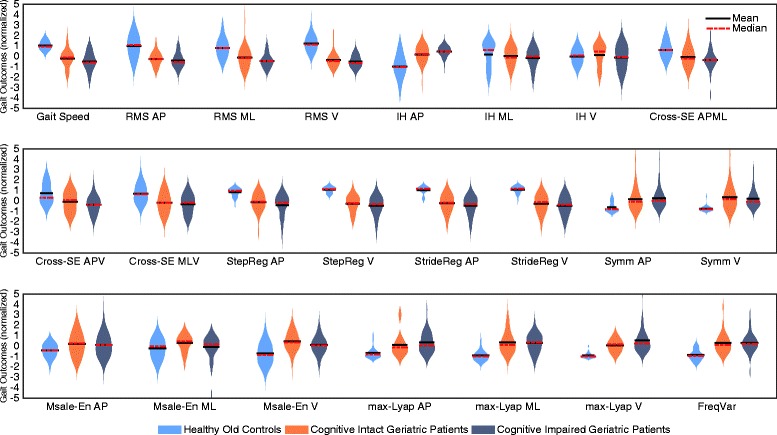
Violin plots based on the kernel density distribution show the distribution of gait outcomes. The violins show gait outcomes for healthy old adults (*n* = 25), cognitive intact (*n* = 31), and cognitive impaired geriatric patients (*n* = 36) during single-task walking. A more compact and less elongated kernel denotes greater density and homogeneity across gait outcomes. Black and dashed red lines indicate mean and median values, respectively. Outcomes are standardized to unit variance for plotting purposes only. RMS = Root Mean Square; IH = Index of Harmonicity; Cross-SampEn = Cross Sample Entropy; Mscale-En = Multi-scale Entropy; max-Lyap = maximal Lyapunov Exponent; FreqVar = Frequency Variability; AP = Anterior-Posterior; ML = Medio-Lateral; V = Vertical

The interpretation of the above results necessitates the direction of the relationship between gait outcomes and participant group. Violin plots show the distribution of the 23 normalized gait outcomes for the three groups during single-task walking (Fig. 1). Both geriatric patient groups walked slower than healthy old adults. In addition, geriatric patients presented with smaller amplitude magnitude (RMS), more synchronization of trunk accelerations (Cross-Sample Entropy), less regularity and symmetry (step and stride regularity and symmetry), less stability (*λ*_max_), and more stride variability (Frequency variability). Gait smoothness (Index of Harmonicity) and gait predictability (Multiscale Entropy) were comparable for geriatric patients and healthy old adults, and showed large kernel sizes. As visible from the violin plots, differences between cognitive intact and cognitive impaired geriatric patients were small for individual gait outcomes.

### Discrimination of groups

**Fig. 2:**
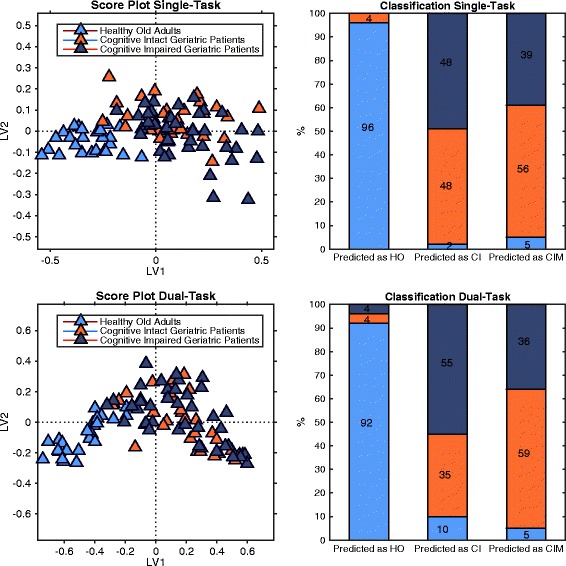
Score plots and classification accuracy. Score plots (*left panel*) visualize the individual participant scores and shows the relationship between gait outcomes and participants of each group with respect to the first two Latent Variables for single-task (*upper panel*) and dual-task walking (*lower panel*). Healthy old adults present in a sharply separated cluster, while 57 and 64% of geriatric patients with cognitive intact (CI) and cognitive impaired (CIM) geriatric patients are misclassified for single- and dual-task, respectively (*right panel*)

For single- and dual-task walking, 24 (96%) and 23 (92%) of the 25 healthy old controls were correctly classified based on the gait outcomes, respectively. Fifteen (48%) and 11 (35%) of 31 cognitive intact geriatric patients were correctly classified during single- and dual-task walking. Fifteen (38%) and 14 (36%) of 39 cognitive impaired geriatric patients were correctly classified based on respectively single- and dual-task walking (Fig. 2). The multivariate models for single- and dual-task conditions were comparable in terms of discriminative ability (VIP-scores), and classification accuracy.

## Discussion

We examined gait characteristics and their discriminative power in healthy old adults and geriatric patients with- and without cognitive impairment. Twenty-three accelerometry-based gait outcomes were calculated while subjects walked for 3 min at habitual speed with and without a cognitive dual-task. Gait outcomes related to speed, regularity, predictability, and stability of trunk accelerations revealed with the highest discriminative power (VIP > 1), and were thus most important in the identification of the three groups of old adults in terms of their gait. Despite the correct classification of a high proportion of healthy old adults (96 and 93% for single- and dual-task, respectively), the classification of geriatric patients with and without cognitive impairment was poor: 57% (single-task) and 64% (dual-task) of the patients were misclassified. We discuss gait characteristics for the three groups, and the lack of discrimination between geriatric patients with and without cognitive impairment.

Gait speed and speed-related outcomes (e.g., RMS, AP Index of Harmonicity) were important in the characterization of groups. Geriatric patients walked substantially slower (0.81 m/s) than healthy old adults (1.20 m/s) but gait speed was similar in patients with (0.88 m/s) and without (0.81 m/s) cognitive impairment. These values compare well with normative gait speed data for healthy old adults [31] and patients with cognitive impairment [19]. In general, age-related gait slowing results from a decline in neuromuscular and neurophysiological functioning that for example engenders sarcopenia and a slower reaction time [1]. Considering geriatric patients’ higher age compared with healthy old adults, the slower gait speed was expected.

With regards to gait regularity, predictability, and stability, gait outcomes in ML and V direction were particularly important, as indicated by the large VIP-scores. Geriatric patients vs. healthy old controls walked less regular, less predictable, and less stable. Especially, gait control in ML direction is crucial in maintaining balance and gait alterations in this direction have been associated with dynamic instability, numerous pathologies, as well as with adverse life-events such as falling [8]. The decline in gait regularity and stability in geriatric patients may be related to a decline in executive functioning. Because imaging studies reported associations between the activation of wide brain networks and gait speed, especially in cognitively impaired old adults, gait is far from an automatically controlled motor task and involves cognitive functioning [32]. In particular, reductions in executive function may result in inaccurate control of limb movements and diminished feed-back that caused gait irregularity and instability [33]. For instance, differences in step and stride regularity have been suggested to reflect differences in the motor control of propulsion and braking phases of gait [7], a process highly depending on lower limb control and accurate feedback mechanisms. In support of this explanation, structural and functional neuroimaging data suggest that prefrontal brain areas (the areas executive functions are predominantly located) are most susceptible to age-related decline [34].

With respect to the classification accuracy, the gait outcomes revealed close to perfect classification of the healthy old group (96 and 92% for single- and dual-task, respectively). However, the discrimination between geriatric patients with and without cognitive impairment was poor, with 57% (single-task) and 64% (dual-task) of the patients misclassified. This finding was unexpected, as the cognitively impaired vs. cognitive intact geriatric patients scored significantly lower on global cognition (3.5 points lower MMSE score), and on executive and memory functioning. Furthermore, an additional cognitive stressor in the dual-task condition did not improve the discrimination between the geriatric patient groups. Our data are in contrast to most previous studies that examined gait in older adults with and without cognitive impairment (see [35] for a recent review), and does not underscore the idea that cognitive impairment can be identified based on gait performance alone. A general finding from cross-sectional studies revealed a gait slowing in patients with MCI as compared to cognitively healthy older adults [20, 36–40], with a slowing up to 0.31 m/s in patients with MCI compared to age-matched controls [19]. Because cognitive impairments are strongly associated with gait slowing [19, 40], we also expected but found only 0.07 m/s additional gait slowing in our patients with cognitive impairment. Subject characteristics may account for discrepancies between our and previous studies. The average age in the above studies ranges from 62 to 79, while our geriatric patients with cognitive impairment were 82 years on average. Our patients attended a geriatric outpatient clinic, indicating that they suffered from general or more specific declines that required comprehensive assessment and/or treatment. Geriatric patients typically suffer from many clinical conditions (as reflected in the Charlson Comorbidity Index; CCI = 1.8) that are known to interact with gait, such as sarcopenia [17], thoracic kyphosis [10], polypharmacy [18]). Although the number and severity of comorbidities remains hard to compare because a clear definition is lacking, comorbidities are often not reported in the literature. In addition, the results of cognitive impaired patients (with comorbidities) are frequently compared to considerably more healthy controls. We suggest that the effects of comorbidities in our frail geriatric patients sum to a level that causes a ‘floor-effect’, so that when cognitive impairment adds to the symptoms, gait does not deteriorate any further, even if tested under dual-task walking.

Our data are in line with a recent population study that concluded that a slow baseline gait speed was only modestly related to future cognitive decline, and provided no early marker of clinical progression from MCI to dementia [41]. Another recent study reported a lack of gait differences between frail and cognitively impaired old adults during single- or dual-task walking [42]. The latter study quantified gait kinematics derived from lower trunk accelerations in old adults aged >75 who meet Frieds’ criteria for frailty. Despite the relatively short walking distance (5 m), they found that the gait kinematics were highly sensitive to distinguish frail groups and healthy old controls, but not to distinguish frail patients with and without cognitive impairment during either single- or dual-task walking. Nascimbeni and colleagues reported comparable conclusions [43]. Our and previous data thus raise the possibility of a clinical threshold beyond which the use of only gait outcomes to identify cognitive impairment is insufficient. We interpret these findings to mean that: (1) the prediction of cognitive impairment from gait abnormalities may be most effective in early phases of cognitive decline, where the influences of comorbidities on gait are limited. Hence, in frail geriatric patients, (2) the identification of cognitive decline requires a multifactorial approach, including physical, cognitive, pharmacological, and behavioural measures.

Despite the relatively low sample size, which can be considered a potential limitation of the study, we speculate that the outcomes can be generalized to similar population groups (i.e., age-matched healthy old adults and geriatric patients admitted to outpatient clinics). Furthermore, although the impact of comorbidities (e.g., sarcopenia, thoracic kyphosis, polypharmacy) on gait function was similar to both geriatric patient groups (Table 1), those factors could have caused the lack of discriminative ability based on gait function alone. Future studies should take into account those interacting factors by applying multi-factorial analyses, or by studying younger patients (aged 60-70) with cognitive impairment who do not yet present with multiple comorbidities. Finally, the present analysis only focused on gait aspects derived from 3D-trunk accelerations. Future studies are encouraged to study the discriminative ability of for example gait kinetics to identify cognitive impairment. A strength of the present analyses can be found in the fact that we only calculated gait outcomes that are independent of step detection. An accurate, automatic, detection of foot-contact indices from acceleration signals is difficult, and already achieves an error rate of 7.4% in healthy old adults [44]. This error rate is expected to increase with gait slowing and/or shuffling; conditions very common among geriatric patients. Hence, we recommend the use of such an approach in geriatric patients.

## Conclusion

In conclusion, gait outcomes related to speed, regularity, predictability, and stability of trunk accelerations were most important in the characterization of patient groups and revealed with a large discriminative power. Such measures were highly sensitive to discriminate healthy old adults from geriatric patients but could not discriminate geriatric patients with and without cognitive impairment during single- or dual-task walking. Thus, our data suggest that caution is needed to predict geriatric patients’ cognitive status from gait performance alone. We propose that an accurate identification of cognitive impairment requires a multivariate approach that comprises not only a comprehensive gait analysis, but also other physical, cognitive, and behavioural measures.

## Abbreviations

3D: Three dimensional
7MS: 7-Minute Screen
AP: Anterior-Posterior
BMI: Body Mass Index
BTO: Benton’s Temporal Orientation
CCI: Charlson Comorbidity Index
Cross-SampEn: Cross-sample Entropy
ECR: Enhanced Cued Recall
FreqVar: Stride frequency variability
IH: Index of Harmonicity
LV: Latent Variable
ML: Medio-Lateral
MMSE: Mini Mental State Examination
Mscale-En: Multi-scale sample Entropy
PLS-DA: Partial Least Squares - Discriminant Analysis
RMS: Root Mean Square
V: Vertical
VIP: Variable Importance in Projection
*λ*_max_: maximal Lyapunov exponent
