# Erratum to: Gait characteristics and their discriminative power in geriatric patients with and without cognitive impairment

**DOI:** 10.1186/s12984-017-0304-4

**Published:** 2017-09-20

**Authors:** Lisette H. J. Kikkert, N. Vuillerme, J. P. van Campen, Bregje A. Appels, Tibor Hortobágyi, Claudine J. C. Lamoth

**Affiliations:** 1 University of Groningen, University Medical Centre Groningen, Center for Human Movement Sciences, A. Deusinglaan 1, 9700 AD Groningen, The Netherlands; 2 Université Grenoble Alpes, EA AGEIS, Grenoble, France; 3 Institut Universitaire de France, Paris, France; 4 Department of Geriatric Medicine, MC Slotervaart Hospital, Amsterdam, The Netherlands; 5 Department of Medical Psychology and Hospital Psychiatry, MC Slotervaart Hospital, Amsterdam, The Netherlands

## Erratum

The original article [1] contained two errors mistakenly carried forward by the Production team handling this article. The errors were as follows:

1. The names of authors Lisette H.J. Kikkert and Claudine J.C. Lamoth were incorrectly presented due to a misplacement of the ‘C’ initial between the two names.
2. The following statement was omitted from the Funding section in the Declarations:
  “This study was supported by the French National Research Agency in the framework of the “Programme d’Investissements d’Avenir IRT Nanoelec,” grants ANR-10-AIRT-05 and ANR-15-IDEX-02 and Institut Universitaire de France.”

The original article has now been corrected to reflect the correct names of the authors Lisette H.J. Kikkert & Claudine J.C. Lamoth, and the aforementioned funding statement has now been inserted accordingly.
